# Progress towards an optimal specimen support for electron cryomicroscopy

**DOI:** 10.1016/j.sbi.2015.12.007

**Published:** 2016-04

**Authors:** Christopher J Russo, Lori A Passmore

**Affiliations:** MRC Laboratory of Molecular Biology, Francis Crick Avenue, Cambridge CB2 0QH, UK

## Abstract

The physical principles of electron–specimen interaction govern the design of specimen supports for electron cryomicroscopy (cryo-EM). Supports are constructed to suspend biological samples within the vacuum of the electron microscope in a way that maximises image contrast. Although the problem of specimen motion during imaging has been known since cryo-EM was first developed, the role of the support in this movement has only been recently identified. Here we review the key technological advances in specimen supports for cryo-EM. This includes the use of graphene as a surface for the adsorption of proteins and the design of an ultrastable, all-gold substrate that reduces the motion of molecules during electron irradiation. We discuss the implications of these and other recent improvements in specimen supports on resolution, and place them in the context of important developments in structure determination by cryo-EM.

## Highlights

• Physical principles of electron scattering govern the design of specimen supports.
• Radiation-induced motion causes loss of resolution in electron micrographs.
• Specimen supports can now be designed to reduce specimen motion.
• Tailored surfaces in the support allow control of particle distribution and orientation.
• Future developments in support technology will further improve image quality.

## Graphical abstract

**Figure:**
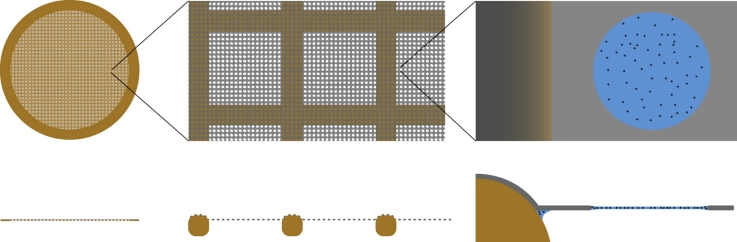

**Current Opinion in Structural Biology** 2016, **37**:81–89

This review comes from a themed issue on **Macromolecular machines and assemblies**

Edited by **David Barford** and **Karl-Peter Hopfner**

For a complete overview see the Issue and the Editorial

Available online 14th January 2016

**http://dx.doi.org/10.1016/j.sbi.2015.12.007**

0959-440X/© 2016 The Authors. Published by Elsevier Ltd. This is an open access article under the CC BY license (http://creativecommons.org/licenses/by/4.0/).

## Introduction

After important studies of the damage caused by high-energy electrons to biological specimens [1, 2] and development of methods to compute 3D density maps from 2D projection images [3, 4, 5], the key technological advance that underpins the field of cryo-EM is the vitrification of water [6^••^]. Vitrification rapidly freezes proteins in thin layers of water ice, thus preserving their structures in a native environment for imaging. The device most often used to support thin layers of ice comprises an amorphous carbon foil suspended across a metal mesh grid [6^••^]. The carbon foil is perforated with holes of order one micrometer in diameter. Biological specimens suspended across the holes are frozen such that the water surrounding them enters an amorphous solid phase, nearly identical to motionless liquid water, which preserves the arrangement of the molecules as they were just before freezing [7^••^].

When irradiated with the electron beam, vitrified biological specimens move and build up semi-static charge long before they are destroyed by the high energy electrons; this blurs the micrographs and limits their resolution. Although this movement has been known since the early days of cryo-EM and many previous studies contributed to understanding its origin [8••, 9, 10, 11, 12, 13, 14, 15•], it was only with the recent advent of direct electron detectors that we have been able to quantify specimen movement with sufficient accuracy to begin to delineate the physical basis of radiation-induced movement [16•, 17•, 18, 19, 20•]. This has revealed that much of the movement is due to the support itself [21^••^]. In this review, we discuss the physical requirements of cryo-EM specimens and consider how supports have improved since Dubochet and colleagues first demonstrated vitrification. This technological progress has, and will continue to facilitate faster and easier data collection and higher resolution images.

## Physical requirements of cryo-EM specimens

**Figure 1:**
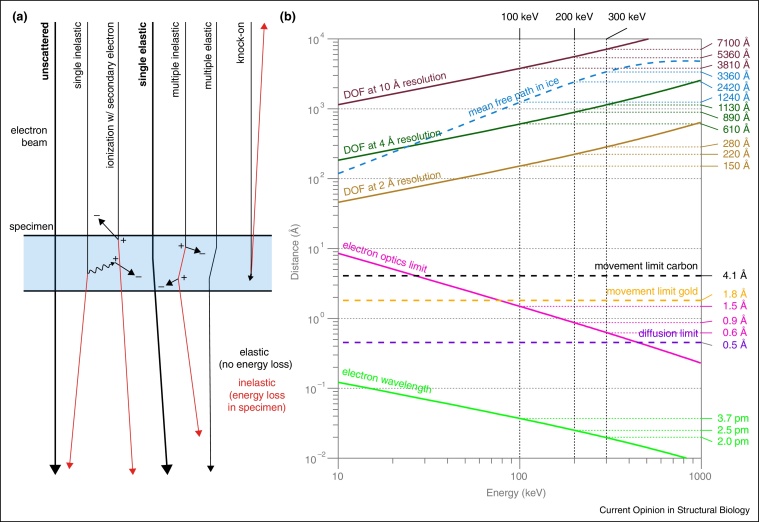
Physical constraints on specimen design in cryo-EM. Diagram **(a)** of high-energy electron scattering in a thin layer of ice, with types of events shown in order of decreasing probability from left to right. Only the unscattered and single elastic scattering events (bold) contribute to typical phase contrast imaging; the remainder damage the specimen (inelastic) or contribute noise to the image. The relative probability of these events is described by their scattering cross sections, whose sum is closely related to the total mean free path, shown in **(b)**. Several other physical parameters that constrain specimen design in cryo-EM are plotted versus energy in (b). Unlike for light microscopy, neither the electron wavelength (light green line) nor the lens optics (pink line, chromatic aberration) limit resolution. Instead, specimen movement during imaging (black dashed line, information limit for moving particles without motion correction on Quantifoil supports) and information content in the individual images limits practical resolution. High-speed detectors can be used to compensate for specimen motion (to move below black dashed line) and new supports reduce movement (gold dashed line, information limit on all-gold grids). Cryo-EM is now starting to approach the information limits imposed by the optics of the microscope (pink line) and the diffusion of the particles within the vitrified ice (purple dashed line, 1 MDa particles). The thickness of the specimen is limited by the total mean free path in ice (blue dashed line), and the depth of field (DOF) at a particular resolution caused by curvature of the Ewald sphere. Theory after [22, 23, 24••, 25, 26, 27, 21••, 28]; see Appendix A.

The interactions of high-energy electrons with solid materials govern specimen design for transmission electron microscopy (EM). The theory of electron specimen interaction [29] was established long before the technology to prepare native biological specimens was developed [7^••^]. Since phase contrast is the imaging mechanism that provides the most information from the sample [24••, 30], specimens for single particle cryo-EM must be designed to maximise this form of contrast. Specimen design centres on minimising the deleterious effects of inelastic and multiple scattering, which do not contribute to phase contrast and cause damage to the specimen (inelastic), while preserving the elastic and unscattered electrons for the generation of phase contrast (Figure 1). Specimens must be thin because electrons cannot traverse materials that are much thicker than the mean free path of the electron in ice, and the thicker the specimen, the more inelastic and multiple scattering effects will degrade image quality. As shown in Figure 1, the mean free path of electrons in water ice is a few tenths of a micrometer, and increases with energy; it saturates at around 1 MeV due to relativistic effects as the electron approaches the speed of light. Specimen thickness is also limited by the depth of field in the image, which increases with energy (Figure 1). For single-particle EM and electron cryotomography, this limits specimen thickness to less than a micrometer, and for high-resolution even thinner: about 300 Å thick for 2 Å resolution at 300 keV.

Specimens must also be thin for vitrification: water must be cooled to cryogenic temperatures in less than a millisecond to stop the molecules from forming crystals [7^••^]. At atmospheric pressure this requires a thin layer of liquid that is less than about three micrometers thick. Any thicker, and the thermal conductivity of the water itself will prevent the water from cooling fast enough to enter the amorphous phase. The instability of thin aqueous layers still presents challenges to reliable sample preparation [31].

Since the specimen is damaged by inelastically scattered electrons at a rate that is faster than it is imaged by the elastically scattered ones [24^••^], it was essential to develop low-dose techniques and supports that minimise irradiation of the specimen. Unfortunately, while low-dose imaging circumvents the fundamental limit of damage to the specimen, it comes at a price: (a) the images become noisy because there are not enough electrons in the image to resolve high resolution features (∼1000 e^−^/Å^2^ are required for atomic resolution but ∼10 e−/Å^2^ destroy the specimen) and (b) when a specimen is first irradiated, it moves (4 Å or more in the first ∼10 e^−^/Å^2^ according to our measurements [20^•^]), which blurs the images and reduces the resolution.

To overcome limitation (a), many images of identical molecules are taken, which are then aligned with each other and averaged, effectively increasing the dose without increasing the damage [3]. But this technique cannot overcome limitation (b) as the high-resolution information is lost in the movement of the particles. More specifically, the maximum resolution for which information is transmitted from a moving specimen to the image is

(1) $$d_{m}=\frac{\pi r}{\sqrt{6\ln S}}$$

In this formula, *d*_*m*_ is the resolution limit of an image where the particles within it have moved in random directions, but on average by a distance *r* [23]. *S* is the signal to noise ratio required to distinguish a particular feature in the image. Here we make the simplifying assumption that the particle velocity can be approximated as being constant during image acquisition. For a signal to noise ratio of ln *S* = 2 and using previous measurements of average particle movement *r* [20•, 21••], we use Equation (1) to calculate the resolution limits plotted in Figure 1b (dashed lines). Compared to other limits on resolution, it is clear that movement is more limiting than either the wavelength of the electron or the optics of the microscope. This illustrates another important improvement in cryo-EM due to the development of direct-electron detectors. By splitting the micrographs in time into movies, tracking the movement of the particles and then compensating for the movement using image correction algorithms, the effective particle movement, *r*, can be reduced [17•, 18, 19, 32]. This lowers the resolution limit, *d*_*m*_, imposed by that movement and accounts for the improved resolution with direct electron detectors versus film which cannot be explained by increased detector efficiency alone.

The buildup of charge on the specimen can induce physical movement of the molecules relative to the microscope, but it can also cause a virtual movement of the particle images by deflecting the image forming electrons [33•, 15•]. This imposes an information limit on the images that is equivalent to the one described above; only now it is the apparent movement in the specimen or variation in defocus across the field of view that blurs the image. More accurate measurements of charging effects are required to quantify this form of blurring, and thus delineate how the electrical properties of the specimen and its support structure limit resolution.

## Supports

### Geometry

**Figure 2:**
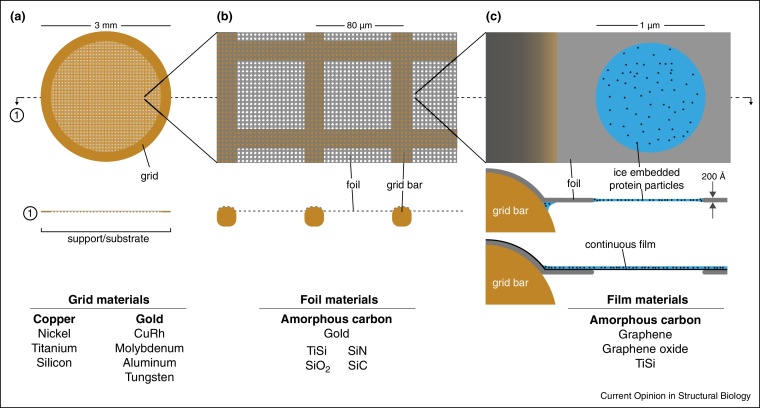
Design of cryo-EM specimen supports. Top view and section diagrams of typical specimen support geometries, comprising a perforated foil on a metal mesh grid. Sometimes an additional thin continuous film is added to the foil to change its surface properties. Three different magnifications are shown **(a)**–**(c)** along with lists of materials used for each component of the support. The most commonly used materials are in bold.

To satisfy the physical constraints discussed above, support designs for biological cryo-EM have converged on a geometry that comprises a perforated foil suspended across a 3 mm grid (Figure 2). Macromolecules in vitrified aqueous solution are suspended across the holes. This use of perforated (not continuous) foils means that samples can be imaged without additional background signal from the support material. This geometry also allows focusing and other parameters to be set using an adjacent area of the support foil. Perforated foils can have holes with a random size distribution, in an irregular arrangement (holey or lacey grids) [34, 35, 36]. More recently, foils with regular arrays of holes of controlled size have been made using micro-fabrication techniques (e.g. Quantifoils® or C-flats^TM^ [37•, 38, 39, 40]), allowing more reproducible specimen preparation and imaging, and facilitating easier low-dose data collection.

### Support development

Some of the first supports used for biological electron microscopy were a mesh made of copper wire [41] or a disc of metal foil with a pinhole [42, 43]. Smaller specimens were supported by adding thin layers of nitrocellulose (collodion) or plastic (e.g. formvar) to the grid [44], but these foils had poor stability and conductivity. Soon, the plastics were replaced by amorphous carbon [45] which was more stable and electrically conductive. Other techniques were developed to manufacture perforated plastic films [34], and coat them with carbon or metal to improve their stability [35].

Since the development of vitrification methods [6^••^], metal grids with perforated amorphous carbon foils have been the support of choice. The most popular grid material has historically been copper, but any metal that can be used for electrodeposition can be made into a grid (Figure 2a). Grids are specified by the pitch of the mesh, usually in the unfortunate but ubiquitous imperial units of lines per inch. Grids of 200–400 lines per inch have squares that are 130–60 μm across, and offer a compromise between stability and imaging area. Carbon is relatively electron transparent (due to its low scattering cross-section) and it is straightforward to manufacture it into perforated foils. Nevertheless, amorphous carbon has limitations. Supports with a copper or gold grid and perforated amorphous carbon foil move during electron irradiation by 200–400 Å perpendicular to the plane of the support [21^••^]. One reason for this is a lack of tension in the carbon foil after cooling. Owing to differing thermal expansion, carbon shrinks less than the most common metals (copper, gold) used for the grid. This leads to ‘cryo-crinkling’ of the carbon [46, 47, 48], which adds a compressive force on the foil and resulting in increased specimen movement [49]. By making the thermal expansion coefficient of the grid less than that of the foil (e.g. molybdenum or tungsten versus carbon), one can minimise crinkling [47, 50, 51]. Still, the physical properties of amorphous carbon are variable (expansion coefficients can vary by a factor of four [52]) so controlling the coefficient mismatch remains challenging. Simply increasing the thickness of the carbon foil reduces radiation-induced motion [48, 49] but carbon has other disadvantages. It is a semiconductor [49, 53] and has poor conductivity compared to most metals, which may contribute to charging of the specimen. Also, physical and chemical changes occur in carbon foils upon electron irradiation [49, 54].

Several alternative foil materials have been used instead of amorphous carbon. Some improvement was observed by coating a carbon foil with gold or titanium-silicon [55]. Pure amorphous titanium-silicon foils have increased conductivity and mechanical strength compared to amorphous carbon [14, 56]. This improves images of 2D-crystals, but their use for single-particle structure determination has not been demonstrated. Doped silicon carbide foils on a silicon frame (Cryomesh^TM^) are flatter and more rigid but are also fragile and difficult to use in practice [57].

**Figure 3:**
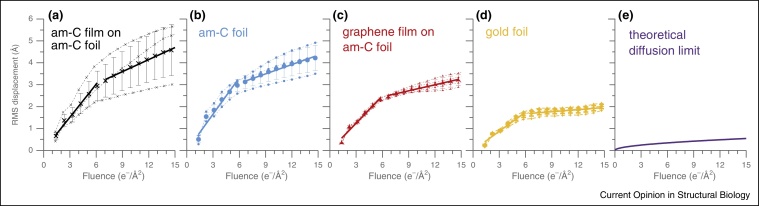
Reducing movement of biological specimens to the physical limits. Electron radiation induced movement of ribosomes was measured on different supports under the same irradiation conditions **(a)**–**(d)**. Ribosomes imaged on amorphous carbon (am-C) supports (a,b) show a large degree of movement during irradiation. Replacing the thin amorphous carbon film (a) with graphene (c) reduces the movement and improves reproducibility. Making the entire support from gold reduces the movement to less than 2 Å in a typical micrograph, (d). Further developments are required to reduce the radiation induced movement to the theoretical limit set by pseudo-diffusion of the particles in the ice **(e)**. Panels (a–d) reproduced from [20•, 21••], panel (e) calculated with the Stokes-Einstein equation using the water diffusion coefficient measured in [28]. Values for these curves at 15 e^−^/Å^2^ are used to calculate the information limits in Figure 1.

We recently showed that manufacturing the grid and the foil out of a single material, gold, overcomes many of these limitations [21^••^]. First, there is no mismatch in thermal expansion so the grid and foil shrink uniformly upon cooling, eliminating any additional compressive stress on the foil. Second, gold is highly conductive (more conductive than TiSi and SiC films), even at liquid nitrogen temperatures [49]. Third, the secondary electron yield of gold is high and this may help neutralise accumulated charge in the ice that distorts images. These supports reduce movement (Figure 3) and improve the resolution of electron cryomicrographs and cryotomograms [21••, 58].

All-gold supports can be used with the same methods as standard Quantifoil supports, are simple to produce in the laboratory and are also commercially available (UltrAuFoil®).

### Protein interactions with surfaces

The physical constraints for EM supports require that macromolecules are embedded in a thin layer, so they are necessarily in close proximity to two surfaces. This configuration is problematic since proteins can interact strongly, and in complicated ways, with these surfaces [59]. Surface interactions are important in cryo-EM specimen preparation because the surface area to volume ratio of the suspended water is large. The ratio of a 1 μm hole filled with 100 Å thick ice is five orders of magnitude greater than that of a 1 μL spherical drop of water. It is therefore not surprising that proteins often denature during specimen preparation. Proteins can be attracted to the support foil and excluded from the thin layer of suspended water. Any surface, including the air–water interface, can induce preferential orientation of molecules and this will be different for every specimen. All of these factors can limit structure determination in practice.

Most support surfaces are hydrophobic and are made more hydrophilic by surface modification techniques to improve their wettability. This is typically achieved by exposing the support surface to a low-energy plasma which is created by the ionisation of a low-pressure gas [60, 61]. Ions from the plasma interact with the surfaces to remove contamination and render them hydrophilic. This can be done using residual air (glow discharging) or under more controlled and defined conditions (e.g. oxygen, argon, hydrogen) [39, 20•]. Other molecules (e.g. amylamine [54, 60]) can be introduced during treatment to alter the surface properties, changing how proteins interact with them. Surfactants (e.g. detergents or phospholipids) [7^••^] or regular arrays of small proteins [62] can also be used to change water interfaces and to modify their interaction with proteins. Surfactants may improve the stability of thin water layers and allow more control of ice thickness [31]. Still, the specific effect of a particular surfactant is difficult to predict and therefore requires trial and error or systematic screening.

A thin continuous film of amorphous carbon can be placed over the perforated foil (Figure 2c) to control one of the two surfaces and improve particle distribution and orientation. Since particles adsorb to the carbon, lower solution concentrations can sometimes be used. In addition to the problems described above, imaging molecules over amorphous carbon adds a significant amount of background noise. To address these shortcomings, several alternative support films have been proposed, including titanium-silicon, carbon nanomembranes and other forms of nanoscale carbon [14, 63, 64].

Interestingly, single crystals of atomically thin graphite were proposed as superior support films for electron microscopy more than fifty years ago [65, 66, 67]. With the discovery of graphene [68], and the development of methods for its large-scale chemical synthesis [69], this idea has been revisited in the context of cryo-EM. Graphene has a higher conductivity and mechanical strength, and lower electron scattering cross-section than any other atomically thin film, making it theoretically ideal as a support film. Methods for the transfer of chemically synthesised graphene onto EM supports were first developed for non-biological specimens [70]. Still, pristine graphene is hydrophobic and needs to be modified for use with aqueous biological specimens. A fully oxidised form of graphene, graphene oxide, demonstrated the feasibility of using graphene derivatives for cryo-EM [71^•^]. Subsequent measurements showed that pristine graphene contributes no background signal at all, in the spatial frequencies of interest to structural biology [72•, 20•]. This is in contrast to the significant background signal from graphene oxide and thin layers of amorphous carbon [72^•^]. Several other graphene modifications have been used to render it more hydrophilic. These include application of protein solution to the bottom side of a graphene-covered support, evaporation of amorphous carbon on the graphene, and glow-discharging [73, 74].

Partial hydrogenation increases the hydrophilicity of monolayer graphene without damaging the graphene lattice and without increasing background signal [20^•^]. To achieve controlled, partial hydrogenation, graphene is treated with a low-energy hydrogen plasma. This removes contamination that contributes to background signal and, importantly, the amount of hydrogenation can be used to tune protein density on the film. Imaging of ribosomes demonstrated that radiation-induced motion of particles is reduced on graphene (Figure 3c) and high resolution 3D structures can be obtained [20^•^].

## Future perspectives and conclusions

More reproducible support manufacturing and robotic control of humidity, temperature and blotting during plunge freezing have simplified the process of generating vitreous ice [75]. Still, there is much trial-and-error during specimen preparation and often the microscopist screens many grids before finding one with suitable ice thickness, appropriate protein distribution within the holes, and sufficiently random particle orientation. Automation is one approach to improving specimen preparation, for example, using inkjet deposition [76, 77]. This includes the development of time-resolved methods that can trap specific and non-equilibrium molecular states [78, 79, 80].

Tailoring the surfaces of the support and development of screening tools could allow rapid and reproducible testing of conditions to facilitate structure determination of any protein. To achieve this, new surface treatments, functionalisation of continuous films, and self-assembled monolayers may allow control of surface–protein interactions and the stability/thickness of thin water layers [31, 81, 82, 83]. When a continuous film is required to tune particle distribution and orientation, partially hydrogenated graphene is currently the best choice, particularly for smaller molecules where it is important to minimise background noise. Future work in the authors’ labs will focus on tuning graphene to allow better control of protein orientation and to combine it with all-gold supports.

Ideally, the support will ensure that the specimen moves much less than the resolution of the microscope used to image it, inhibit the buildup of charge that may distort images, and afford control over the position, orientation and distribution of the specimen in the field of view. All-gold supports improve upon previous technology by substantially reducing radiation-induced motion, but further development is needed to reduce this movement to the theoretical limits (Figure 3e) and achieve an ideal support structure. To reach this goal, support materials, surfaces and geometry will need improvement in conjunction with new methods for specimen preparation. The optimum specimen support will maximise the high-resolution information content available in each image and enable atomic resolution structure determination with a few thousand particles from a handful of micrographs.

## References and recommended reading

Papers of particular interest, published within the period of review, have been highlighted as:

• of special interest
•• of outstanding interest

## Figures and Tables

**Table:** 

Description	Formula	Reference(s)	Notes
Electron wavelength	$\lambda =\mathrm{hc}/\sqrt{2{\mathrm{EE}}_{0}+E^{2}}$	[27]	
Chromatic aberration limit	$d_{c}=\sqrt{\pi \Delta \lambda /2}$	[23]	
Inelastic mean free path	$\Lambda_{i}=C/\beta^{2}\ln(\beta^{2}(E+E_{0})/\bar{E})$	[22]	†
Depth of field	$R=\sqrt{1.4/(t\lambda )}$	[26]	66° phase error‡

## Formulae for Figure 1

The mathematical formulae used to generate the plots in Figure 1, are tabulated below.

*E* is the electron energy, *E*_0_ is the electron rest energy (511 keV), *t* is the specimen thickness, *h* is Planck's constant, *β* is the ratio of the electron speed to the speed of light, *C* is the empirical constant determined from the fit and $\bar{E}$ is the mean energy loss, assumed to be 20 eV.

†—Theoretical formula for mean free path was fit to best available experimental measurements of the inelastic mean free path in amorphous water ice, 2030 ± 330 Å [25]. Using the inelastic value will slightly underestimate the total mean free path but since the elastic and inelastic cross sections scale together in the energy range of interest and given the error in the measurement, it is reasonable for the purposes of this discussion.

‡—Several definitions of depth of field are discussed in the literature for high resolution phase contrast, all of which rely on somewhat arbitrary criteria for defining the depth limits. Most use the weak phase approximation, which has questionable validity in predicting resolution limitations due to specimen thickness but has proved useful in describing high resolution phase contrast [for a discussion see Ref. 84]. With this in mind, we took the depth of field due to curvature of the Ewald sphere described by DeRosier [26], as a reasonable, if perhaps somewhat optimistic, estimate of depth of field for the purposes of this review.
